# Integrated Analysis of COX-2 and iNOS Derived Inflammatory Mediators in LPS-Stimulated RAW Macrophages Pre-Exposed to *Echium plantagineum* L. Bee Pollen Extract

**DOI:** 10.1371/journal.pone.0059131

**Published:** 2013-03-08

**Authors:** Eduarda Moita, Angel Gil-Izquierdo, Carla Sousa, Federico Ferreres, Luís R. Silva, Patrícia Valentão, Raúl Domínguez-Perles, Nieves Baenas, Paula B. Andrade

**Affiliations:** 1 Rede de Química e Tecnologia (REQUIMTE)/Laboratório de Farmacognosia, Departamento de Química, Faculdade de Farmácia, Universidade do Porto, Porto, Portugal; 2 Research Group on Quality, Safety and Bioactivity of Plant Foods, Department of Food Science and Technology, Centro de Edafología y Biología Aplicada del Segura (CEBAS), Consejo Superior de Investigaciones Científicas (CSIC), Murcia, Spain; Instituto de Química, Universidade de São Paulo, Brazil

## Abstract

Oxidative stress and inflammation play important roles in disease development. This study intended to evaluate the anti-inflammatory and antioxidant potential of *Echium plantagineum* L. bee pollen to support its claimed health beneficial effects. The hydromethanol extract efficiently scavenged nitric oxide (^•^NO) although against superoxide (O_2_^•−^) it behaved as antioxidant at lower concentrations and as pro-oxidant at higher concentrations. The anti-inflammatory potential was evaluated in LPS-stimulated macrophages. The levels of ^•^NO and L-citrulline decreased for all extract concentrations tested, while the levels of prostaglandins, their metabolites and isoprostanes, evaluated by UPLC-MS, decreased with low extract concentrations. So, *E. plantagineum* bee pollen extract can exert anti-inflammatory activity by reducing ^•^NO and prostaglandins. The extract is able to scavenge the reactive species ^•^NO and O_2_^•−^ and reduce markers of oxidative stress in cells at low concentrations.

## Introduction

**Figure 1:**
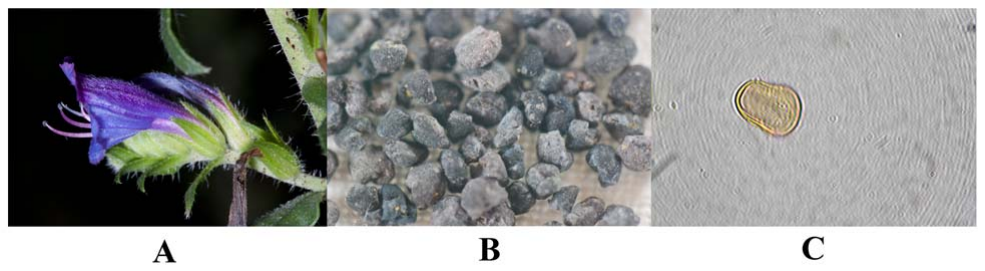
*E. plantagineum* L. (A) Flower. (B) Bee pollen pellets. (C) Pollen grain (1000× magnification).

Bee pollen, used as a dietary supplement, is promoted as a health food with a wide range of nutritional and therapeutic properties. *Echium plantagineum* L., native to southern Europe, is known by its characteristic flower with a purple corolla and two protruding stamens and one stigma, thus being commonly known as purple viper's bugloss (Figure 1). This species is visited by bees especially at the end of the floral period and dark blue bee pollen pellets (Figure 1) are then collected and commercialized [1]. Furthermore, pollen from *Echium* species is commonly found in honeys [2].

From a nutritional point of view, pollen is a good source of proteins and essential amino acids. The total amino acids content in *E. plantagineum* pollen can reach 32% [3]. Other metabolites previously described in *E. plantagineum* are phenolic compounds [1], [4].

Phenolic compounds are thought to be the active ingredients in many dietary plants and traditional medicines used for the treatment of disorders related to oxidative stress and inflammation [5]. There is increasing evidence that phenolic compounds present in natural foods may reduce the risk of chronic diseases, such as cancer, inflammation, cardiovascular and neurodegenerative disorders [6].

Although the inflammatory response is a defence mechanism against infection or injury, sustained inflammation is a pathological condition. Over-expression of several pro-inflammatory enzymes and reactive species, like superoxide (O_2_^•−^) and nitric oxide (^•^NO) radicals, are produced during inflammation [7]. ^•^NO is generated from oxygen and L-arginine by inducible nitric oxide synthase (iNOS), this enzyme being up-regulated during the inflammatory process [8].

Other important mediators over-expressed during inflammation are prostaglandins (PGs). PGs are bioactive signalling molecules derived from cyclooxygenase (COX) and subsequent PG synthase activity on arachidonic acid. Like iNOS, COX-2 is highly up-regulated in response to infection, atherosclerosis and a number of cancers [9]. It plays a pivotal role in the mediation of inflammation, and catalyzes the rate-limiting step in prostaglandin biosynthesis. PG synthases can form important signalling molecules, including PGI_2_, thromboxane A_2_, PGE_2_, PGD_2_ and PGF_2α_ [10]. Thus, inhibiting the activity or expression of these enzymes might be important for cancer and inflammation chemoprevention.

The up-regulation of iNOS and COX-2 during inflammation is controlled by the pro-inflammatory transcription factor NF-κB. Flavonoids that inhibit the induction of this factor are regarded as interesting tools for inflammation control [11], [12].

Besides PGs, isoprostanes (IsoPs), resulting from the free radical-catalyzed peroxidation of arachidonic acid in a COX independent mechanism, exert potent biological activities. They are seen as oxidative stress markers and potentially mediate some of the adverse effects of oxidant injury [13].

Eicosanoids (PGs and IsoPs) determination is currently performed by enzyme-linked immunosorbent assays, but UPLC-MS is more suitable for the simultaneous analysis of multiple eicosanoids that can be involved in inflammatory processes or are markers of oxidative stress. This metabolic approach can successfully be used to determine bioactive molecules that are produced in these complex biological processes [14].

The aim of this study was to assess the anti-inflammatory and antioxidant potential of *E. plantagineum* bee pollen. A hydromethanolic extract was chosen since it is known to be rich in non-coloured flavonoids [4]. Besides, plants contain a complex mixture of compounds that can act in concert to exert a specific bioactivity, more effectively than individual compounds [15].

As far as we know, it is the first time that *E. plantagineum* bee pollen is studied regarding these biological activities.

## Materials and Methods

### Standards and reagents

Lipopolysaccharide (LPS) from *Salmonella enterica* and quercetin-3-*O*-rutinoside were from Sigma-Aldrich (St. Louis, MO, USA) and kaempferol-3-*O*-rutinoside and kaempferol-3-*O*-glucoside from Extrasynthese (Genay, France). Dulbecco's Phosphate Buffered Saline (DPBS), DMEM + GlutaMAX™-I, heat inactivated foetal bovine serum (FBS) and Pen Strep solution (Penicillin 5000 units/mL and Streptomycin 5000 µg/mL) were from Gibco, Invitrogen™ (Grand Island, NY, USA). PGE_1_, PGE_2_, tetranor-PGEM, tetranor-PGAM, 20-hydroxy-PGE_2_, 6-keto-PGF_1α_, 2,3-dinor-6-keto-PGF_1α_, 11β-PGF_2α_, 2,3-dinor-11β-PGF_2α_, tetranor-PGFM, 9,11-dideoxy-9α,11α-epoxymethano-PGF_2α_ (U-44069), 9,11-dideoxy-9α,11α-methanoepoxy-PGF_2α_ (U-46619), 11-dehydrothromboxane B2, 8-iso-PGF_2α_, 2,3-dinor-8-iso-PGF_2α_, 8-iso-15(*R*)-PGF_2α_, 8-iso-15-keto-PGF_2α_, 8-isoprostaglandin F_2α_-d4 and 11-dehydro-thromboxane B2-d4 were from Cayman Chemicals (Ann Arbor, Michigan, USA).

### Sampling

*Echium plantagineum* L. bee pollen sample was provided by bee-keepers in Extremadura Spanish region. The botanical origin was assured by Prof. Paula B. Andrade (Laboratório de Farmacognosia, Faculdade de Farmácia, Universidade do Porto). Pollen was stored protected from light under desiccating conditions to prevent alteration.

### Extract preparation

The extraction was adapted from a method previously described [4]. Bee pollen (0.2 g) was thoroughly mixed with 1 mL methanol:water (7∶3), ultra-sonicated for 1 h, centrifuged at 2,900× g during 10 min (Rotofix 32A, Hettichlab, Germany).

For the HPLC-DAD analysis of phenolic compounds the supernatant was filtered through a 0.45 µm filter.

For the bioactivity assays the supernatant was evaporated under reduced pressure at 40 °C. The residue was dissolved in the appropriate solvent and kept at −20°C until use.

### HPLC-DAD analysis of phenolic compounds

The extract was analysed as described before [4] using a LiChroCART column (250× 4 mm, RP-18, 5 µm particle size, LiChrospher®100 stationary phase, Merck, Darmstadt, Germany). The mobile phase consisted of two solvents: water-formic acid (1%) (A) and methanol (B). Elution started with 30% B and a gradient was used to obtain 40% at 20 min, 50% at 25 min, 60% at 30 min and 80% at 32 min. The flow rate was 1 mL/min, and the injection volume 20 µL. Spectral data from all peaks were accumulated in the range 240–600 nm, and chromatograms were recorded at 350 nm. The data were processed on a Unipoint Software system (Gilson Medical Electronics, Villiers le Bel, France). Peak purity was checked by the software contrast facilities.

Phenolic compounds quantification was achieved by the absorbance recorded at 350 nm relative to external standards. Because standards of all identified flavonoid derivatives were not commercially available, quercetin-3-*O*-neohesperidoside was quantified as quercetin-3-*O*-rutinoside, and kaempferol derivatives as kaempferol-3-*O*-rutinoside, except kaempferol-3-*O*-glucoside, which was quantified as itself.

### Superoxide radical (O_2_^•−^) scavenging in cell free system

The radical was generated by the phenazine methosulphate (PMS)/NADH system and monitored by the reduction of nitroblue tetrazolium chloride (NBT) to formazan [16]. The reaction mixtures consisted of NADH (166 µM), PMS (2.7 µM), NBT (43 µM) and the appropriate extract concentration. All components were dissolved in phosphate buffer (19 mM, pH 7.4). The reaction was conducted at room temperature for 2 min and initiated by the addition of PMS. The absorbance variation was measured spectrophotometrically at 562 nm, in a Multiskan Ascent plate reader (Thermo, Electron Corporation). Five different extract concentrations were tested. Three experiments were performed, in which each concentration was tested in triplicate.

### Nitric oxide (^•^NO) scavenging in cell free system

Nitric oxide was generated from sodium nitroprusside (SNP) and measured by the Griess reagent as before [17]. SNP (6 mg/mL) in phosphate buffer was mixed with different concentrations of bee pollen extract dissolved in the same solvent, and incubated at room temperature for 1 hour under light. After the incubation period the nitrite accumulated in the reaction medium was measured. Based on the Griess reaction, equal volumes of reaction medium and Griess reagent [1∶1 mixture (v/v) of 1% sulphanilamide and 0.1% N-(1-naphthyl) ethylenediamine in 2% H_3_PO_4_] were mixed and incubated for 10 min in the dark, at room temperature. The cromophore was determined in a Multiskan Ascent plate reader at 562 nm. Five different extract concentrations were tested. Three experiments were performed, in which each concentration was tested in triplicate.

### RAW 264.7 macrophages culture and treatments

Macrophage RAW 264.7 cells were from the American Type Culture Collection (LGC Standards S.L.U., Spain) and were kindly provided by Prof. Maria S. J. Nascimento (Laboratório de Microbiologia, Departamento de Ciências Biológicas, Faculdade de Farmácia, Universidade do Porto). They were cultured at 37 °C, in DMEM supplemented with 10% FBS and 2% Pen Strep solution in a humidified atmosphere of 5% CO_2_, as previously reported [18]. Cells were seeded in 48-well plates (150 000 cells/well) and cultured until 80–90% confluence.

Two groups of RAW 264.7 cells were considered: control group (C group) and LPS-treated group (T group). Both C and T groups were cultured in cell media containing growing doses of bee pollen extract (C0 and T0: 0; C1 and T1: 0.5; C2 and T2: 1.0; C3 and T3: 2.1; C4 and T4: 4.2; C5 and T5: 8.3 mg mL^−1^; for viability assays 16.7 and 33.3 mg mL^−1^ were also tested) dissolved in medium containing 0.5% DMSO. Exposure periods were 19 h for viability ^•^NO and L-citrulline assays and 9 h for eicosanoids determination. One µg mL^−1^ of LPS was added to the culture media of T group cells after the first hour of extract exposure and this inflammatory stimulus was maintained during the following 8 or 18 h.

### LDH release

After the treatment period, the culture medium was taken to determine the activity of lactate dehydrogenase (LDH) leaked through cell membranes according to a described procedure [17]. LDH activity was determined at 340 nm. Results are expressed as a percentage of the respective control (with or without LPS). Five independent assays were performed in duplicate.

### MTT reduction

After the incubation period, RAW 264.7 cells were washed with DPBS and then incubated for 30 minutes with 3-(4,5-dimethylthiazol-2-yl)-2,5-diphenyltetrazolium bromide (MTT; 0.5 mg mL^−1^ in DMEM). The extent of the reduction to formazan within the cells was quantified at 510 nm [17]. Results are expressed as percentage of the respective control (with or without LPS). Five independent assays were performed in duplicate.

### ^•^NO in RAW 264.7 cells culture medium

Nitrite accumulated in the culture medium of C and T-group cells was measured after 18 h incubation. This determination was based on the Griess reaction as in “Nitric oxide (^•^NO) scavenging in cell free system” section [17], [19]. Five independent assays were performed in duplicate.

### Determination of L-citrulline

L-citrulline was quantified using the method of Senshu and collaborators [20] with the modifications of Marzinzig and collaborators [21]. Briefly, after 18 h incubation, 100 µL of culture medium of RAW 264.7 cells of C and T-groups were deproteinized with 200 µl of trichloroacetic acid (2.45 M). After centrifugation (10 min; 2,900× *g*), 250 µL supernatant were mixed with 100 µL of reaction mixture containing 40% (v/v) diacetylmonoxime (79 mM in 83 mM acetic acid), 18% (v/v) antipyrine E (47.8 mM in H_2_O) and 42% (v/v) H_2_SO_4_ 7.5 M, and incubated at 96°C for 25 minutes. The solution was then cooled down to room temperature and the absorption was read at 405 nm. Five independent assays were performed in duplicate.

### Extraction of eicosanoids from cells and culture medium

Eicosanoids were determined in the whole cell extracts and in the culture media of RAW 264.7 cells. The culture medium was collected after 9 h exposure, 0.005% BHT (final concentration) was added and the medium was kept at −80°C until eicosanoids extraction. To precipitate the serum proteins present in the culture media 3 mL of methanol/HCl 200 mM was added to 2 mL medium and spun at 10000× *g*, for 5 min.

After removing the culture medium cells were lysed by incubation with lysis buffer (50 mM Tris-HCl pH 8.0, 150 mM NaCl, 1% Triton X-100, containing 0.005% BHT) for 1 h, at 0°C. Lysates were centrifuged at 8,000× *g* for 5 min. The resulting supernatants were kept at −80°C until eicosanoids extraction.

Before eicosanoids extraction one replicate of each culture condition was hydrolyzed using ∼5000 UE mL^−1^ of β-glucuronidase, type H2 from *Helix pomatia* [22].

After samples hydrolysis, both hydrolyzed and non-hydrolyzed supernatants were subjected to solid-phase extraction (SPE) by Strata X-AW cartridge (100 mg 3 mL^−1^) following the procedure of Medina et al. [22]. Target compounds were eluted with methanol and dried using a SpeedVac concentrator.

### UPLC-QqQ-MS/MS analyses of eicosanoids

The separation of eicosanoids in the cell lysates and culture medium was performed by UPLC coupled to 6460 QqQ-MS/MS (Agilent Technologies, Waldbronn, Germany) using the set up of Medina and collaborators [22], [23].

The quantification of the separate eicosanoids detected was performed using the standard compounds PGE_1_, PGE_2_, tetranor-PGEM, tetranor-PGAM, 20-hydroxy-PGE_2_, 6-keto-PGF_1α_, 2,3-dinor-6-keto-PGF_1α_, 11β-PGF_2α_, 2,3-dinor-11β-PGF_2α_, tetranor-PGFM, 9,11-dideoxy-9α,11α-epoxymethano-PGF_2α_ (U-44069), 9,11-dideoxy-9α,11α-methanoepoxy-PGF_2α_ (U-46619), 11-dehydrothromboxane B2, 8-iso-PGF_2α_, 2,3-dinor-8-iso-PGF_2α_, 8-iso-15(*R*)-PGF_2α_, 8-iso-15-keto-PGF_2α_. The 8-isoprostaglandin F_2α_-d4 (contains 4 deuterium atoms at positions 3, 3′, 4, and 4′) and 11-dehydro-thromboxane B2-d4 (contains 4 deuterium atoms at positions 3, 3′, 4, and 4′) were used as internal standards [22], [23].

### Statistical analysis

Statistical analysis was performed using Graphpad Prism 5 Software (San Diego, CA, USA). One-way ANOVA and Bonferroni's test, as post-hoc test, were used to determine the statistical significance in comparison to control. Two-way ANOVA and Sidak's multiple comparisons test were used to determine the interaction between extract and LPS in cell viability and differences in citrulline and ^•^NO release in LPS-stimulated macrophages. Data are expressed as the mean ± SEM. *p* Values of 0.05 or less were considered significant.

## Results

### Phenolic profile

**Figure 2:**
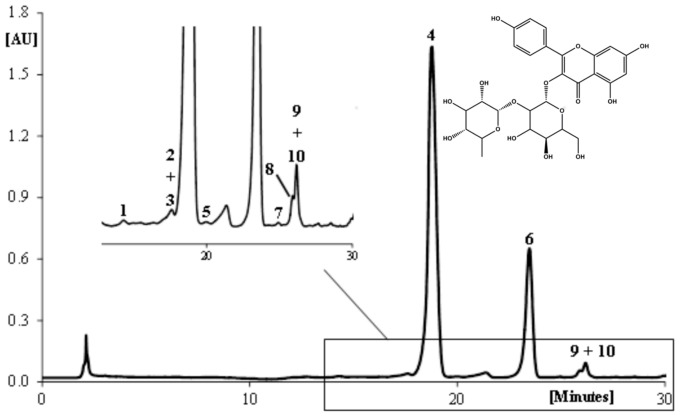
HPLC-DAD chromatogram (350 nm) of phenolic compounds in *E. plantagineum* bee pollen hydromethanolic extract and chemical structure of the main compound, kaempferol-3-*O*-neohesperidoside (4). Other compounds: (1) Quercetin-3-*O*-neohesperidoside; (2) kaempferol-3-*O*-sophoroside; (3) kaempferol-3-*O*-(4′-rhamnosyl)neohesperidoside; (5) kaempferol-3-*O*-neohesperidoside derivative; (6) kaempferol-3-*O*-(3′/4′-acetyl)neohesperidoside; (7) kaempferol-3-*O*-neohesperidoside-7-*O*-rhamnoside; (8) kaempferol-3-*O*-glucoside; (9) kaempferol-3-*O*-rutinoside; (10) kaempferol-3-*O*-(3′/4′-acetyl)-neohesperidoside isomer.

The phenolic profile of *E. plantagineum* bee pollen hydromethanolic extract was established by HPLC-DAD (Figure 2). The chromatogram showed kaempferol-3-*O*-neohesperidoside (**4**) as a major peak, followed by its acylated derivative, kaempferol-3-*O*-(3′/4′-acetyl)-neohesperidoside (**6**), and eight minor peaks, comprising both quercetin and kaempferol derivatives. The content of compounds **4** and **6** was ca. 9 and 3 g kg^−1^, respectively. The other phenolics accounted together for 0.4 g kg^−1^ (Table 1).

**Table 1:** Phenolic compounds from *E. plantagineum* bee pollen extract.

Phenolic compounds		(mg kg^−1^)^a^
**1**	Quercetin-3-*O*-neohesperidoside	24.8±0.1
**2+3**	Kaempferol-3-*O*-sophoroside + kaempferol-3-*O*-(4′-rhamnosyl)-neohesperidoside	120.9±0.1
**4**	Kaempferol-3-*O*-neohesperidoside	8864.1±14.3
**5**	Kaempferol-3-*O*-neohesperidoside derivative	25.8±0.6
**6**	Kaempferol-3-*O*-(3′/4′-acetyl)-neohesperidoside	2988.1±10.0
**7**	Kaempferol-3-*O*-neohesperidoside-7-*O*-rhamnoside	20.7±0.7
**8**	Kaempferol-3-*O*-glucoside	15.1±0.1
**9+10**	Kaempferol-3-*O*-rutinoside + kaempferol-3-*O*-(3′/4′-acetyl)-neohesperidoside isomer	213.6±2.1
	∑	**12273.2**

a Values are expressed as mean ± standard deviation of three determinations.

### Antioxidant activity

**Figure 3:**
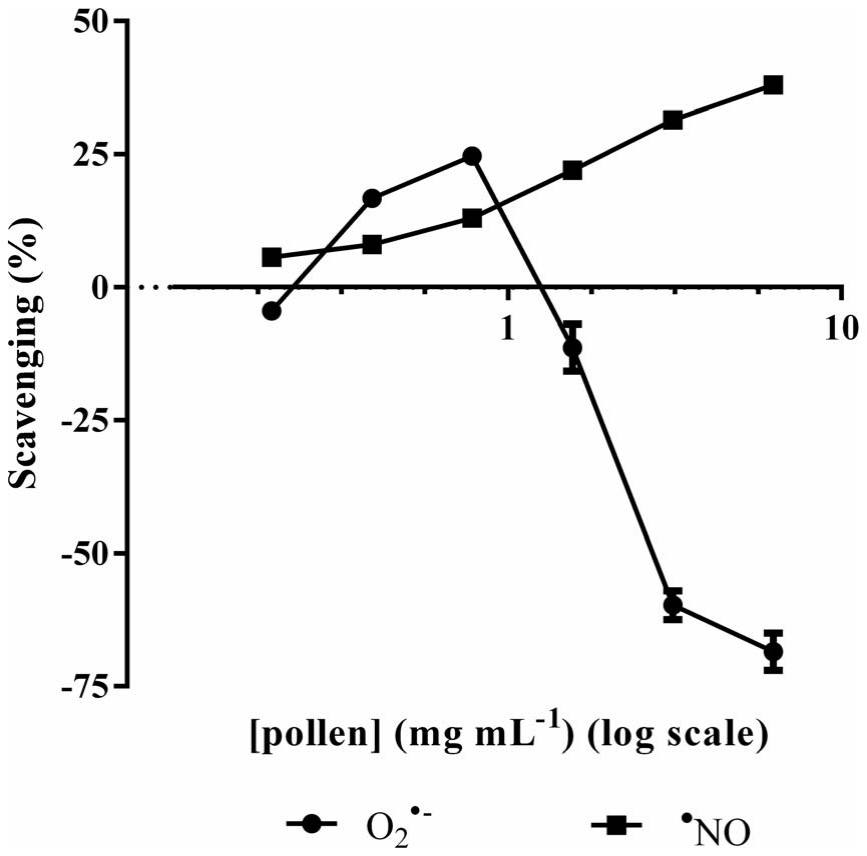
Effect of *E. plantagineum* bee pollen extract in ^•^NO and O_2_^•−^ in cell-free systems. ^•^NO was generated from SNP and measured by the Griess reaction. O_2_^•−^ was generated by the PMS/NADH system and monitored by the reduction of NBT. Results expressed as mean ± SEM of three experiments performed in triplicate.

The hydromethanolic extract of *E. plantagineum* bee pollen scavenged the sodium nitroprusside-generated ^•^NO in a concentration dependent manner (IC_25_ = 1.9 mg mL^−1^) (Figure 3). However, a dual behaviour was seen against O_2_^•−^ generated by the PMS/NADH system: until 0.8 mg mL^−1^ a dose-dependent scavenging effect was observed, but a pro-oxidant response was noticed with higher concentrations (Figure 3).

### RAW 264.7 macrophages viability

**Figure 4:**
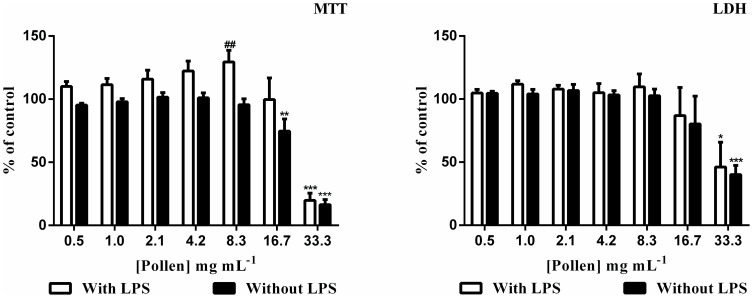
Effect of *E. plantagineum* bee pollen extract in cell viability. RAW 264.7 macrophages were pre-exposed to the extract for 1 h followed by 18 h co-exposition with 1 mg mL^−1^ of LPS. Cell viability was assessed by MTT reduction and LDH release assays. Values show mean ± SEM of five independent assays performed in duplicate. *p<0.05; **p<0.01; ***p<0.001, compared to the respective control; ##p<0.01 LPS-exposed cells compared with cells exposed only to the respective extract concentration.

The extract was not cytotoxic until 8.3 mg mL^−1^ (equivalent to 172 µM of kaempferol and quercetin derivatives). The exposure to 16.7 mg mL^−1^ for 19 h induced a significant decrease in cell viability (*p*<0.01), as assessed by the MTT assay. The cellular viability was further decreased by the extract at 33.3 mg mL^−1^, being statistically significant in both LDH and MTT assays (*p*<0.001) (Figure 4).

Cell treatment with 1 µg mL^−1^ LPS didńt significantly alter cell viability and the results obtained with cells exposed to extract and LPS were similar to the ones found with cells exposed only to the extract (*p*>0.05) (Figure 4). In fact, for the concentration of 8.33 mg mL^−1^ an increase in viability was shown (*p*<0.01).

### ^•^NO and L-citrulline in culture medium

**Figure 5:**
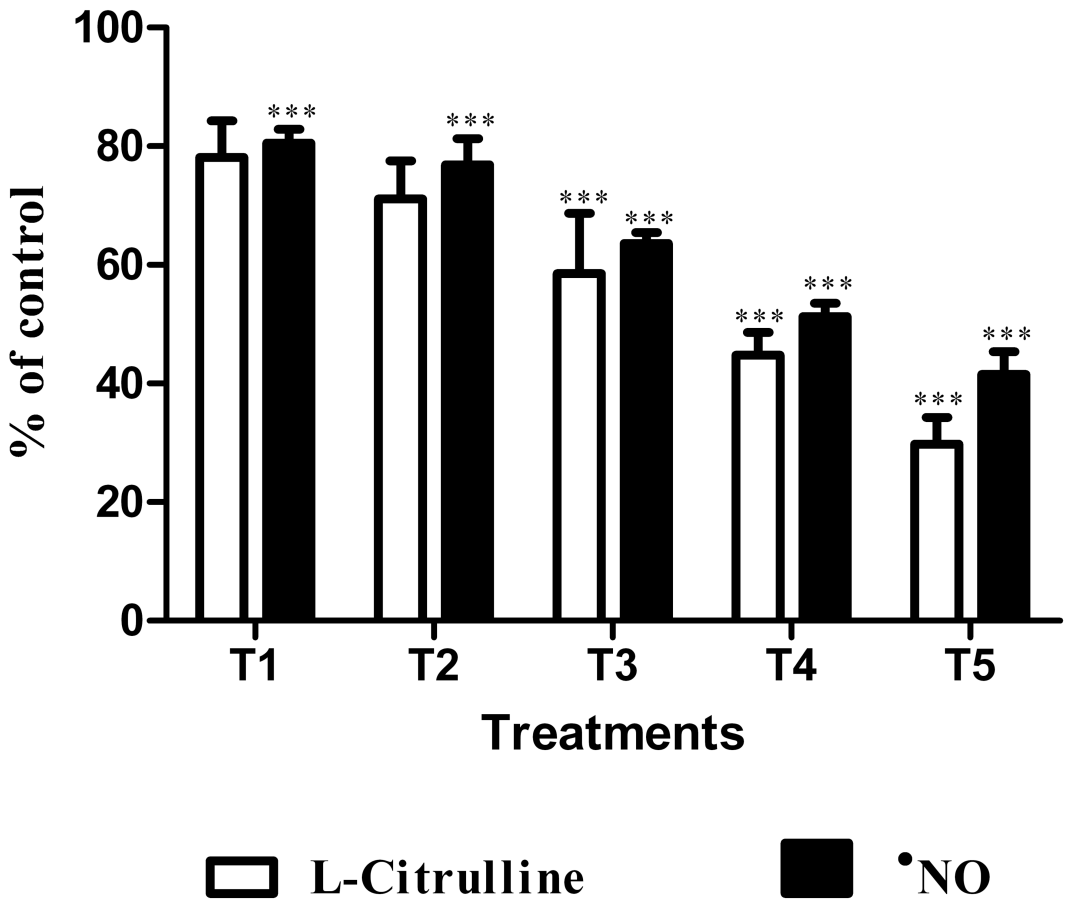
Effect of *E. plantagineum* bee pollen extract pre-exposure in ^•^NO production by LPS-stimulated macrophages. RAW 264.7 macrophages were pre-exposed to the extract for 1 h followed by 18 h co-exposition with 1 mg mL^−1^ of LPS (T-group). Extract concentrations: T0: 0; T1: 0.5 mg mL^−1^; T2: 1.0 mg mL^−1^; T3: 2.1 mg mL^−1^; T4: 4.2 mg mL^−1^; T5: 8.3 mg mL^−1^. Values show mean ± SEM of five independent assays performed in duplicate. ***p<0.001, compared to LPS-only exposed cells.

The levels of ^•^NO and L-citrulline in the culture medium of LPS stimulated cells (T group) after 18 h exposure were 11.8±1.7 and 10.8±0.9 µM, respectively. The levels of these metabolites in the C-group were below the quantification limit. *E. plantagineum* bee pollen extract at non-toxic concentrations dose-dependently decreased ^•^NO in the culture medium of LPS-stimulated cells (*p*<0.001) (Figure 5). Accordingly, the formation of L-citrulline was also inhibited by the extract at all concentrations, being significant for concentrations higher than 2.1 mg mL^−1^ (*p*<0.001) (Figure 5).

### Intracellular eicosanoid levels

**Figure 6:**
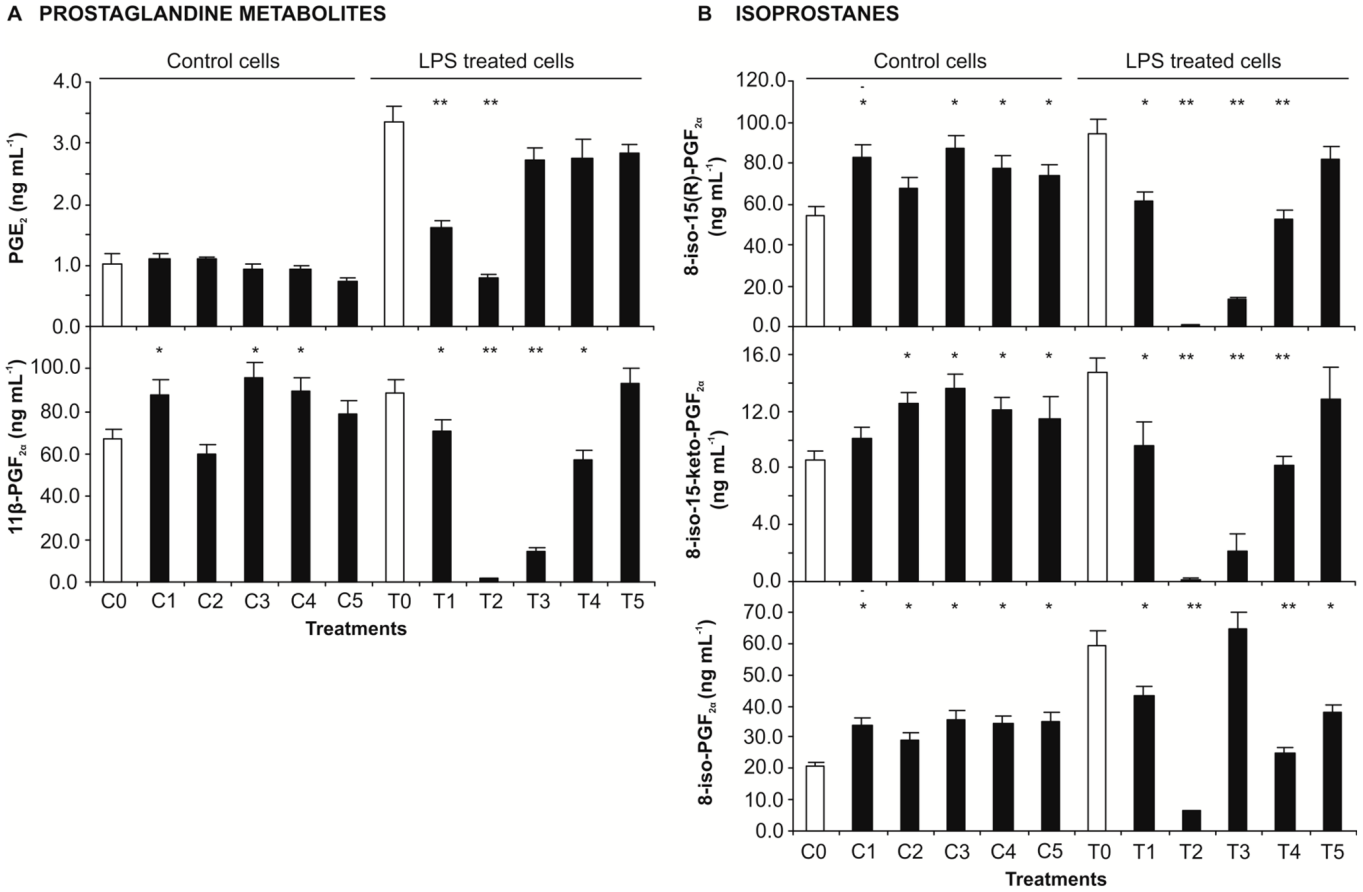
Effect of *E. plantagineum* bee pollen extract pre-exposure in intracellular eicosanoids levels of LPS-stimulated macrophages. Eicosanoids were determined in the whole cell extracts of RAW 264.7 macrophages pre-exposed to the extract for 1 h followed by 8 h co-exposition with 1 mg mL^−1^ of LPS. C group: control group exposed to extract only. T group: extract and LPS co-exposed group. Extract concentrations: C0 and T0: 0; C1 and T1: 0.5 mg mL^−1^; C2 and T2: 1.0 mg mL^−1^; C3 and T3: 2.1 mg mL^−1^; C4 and T4: 4.2 mg mL^−1^; C5 and T5: 8.3 mg mL^−1^. Values show mean ± SEM of two independent experiments performed in triplicate. (A) Prostaglandin and prostaglandin metabolites. (B) Isoprostanes. *p<0.05; **p<0.01 compared to the respective control (with or without LPS).

The levels of prostaglandins and isoprostanes were measured in RAW macrophages whole-cell lysate obtained under mild conditions (Figure 6). Two prostaglandins (PGE_2_ and 11β-PGF_2α_) were quantified both in control cells and in cells exposed to LPS, but higher levels were observed in the latter (Fig. 6A). The levels of PGE_2_ (about 1 ng mL^−1^) in non-stimulated cells were not significantly affected by bee pollen extract. The increased levels of PGE_2_ in LPS-stimulated cells were reduced by the extract at 0.5 and 1 mg mL^−1^ (*p*<0.01), but for higher concentrations weren't significantly different from cells exposed only to LPS (Figure 6A).

In what concerns to 11β-PGF_2α_, the extract showed a tendency to increase the levels of this PGD_2_ metabolite in control cells. In LPS exposed cells the levels of 11β-PGF_2α_ were dose-dependently decreased by the extract at 0.5 and 1 mg mL^−1^, but raised for higher concentrations, being similar to that found in LPS-only exposed cells for the highest extract concentration tested (Figure 6A).

Besides prostaglandins, three isoprostanes (8-iso-15(*R*)-PGF_2α_, 8-iso-15-keto-PGF_2α_ and 8-iso-PGF_2α_) were quantified in the cellular lysate (Figure 6B). Their contents in control cells were increased by the extract. LPS by itself raised the levels of isoprostanes inside the cell and, in general, bee pollen extract decreased IsoPs below the basal level at 1 mg mL^−1^. Higher extract concentrations raised IsoPs levels (Figure 6B).

### Eicosanoid levels in culture medium

**Figure 7:**
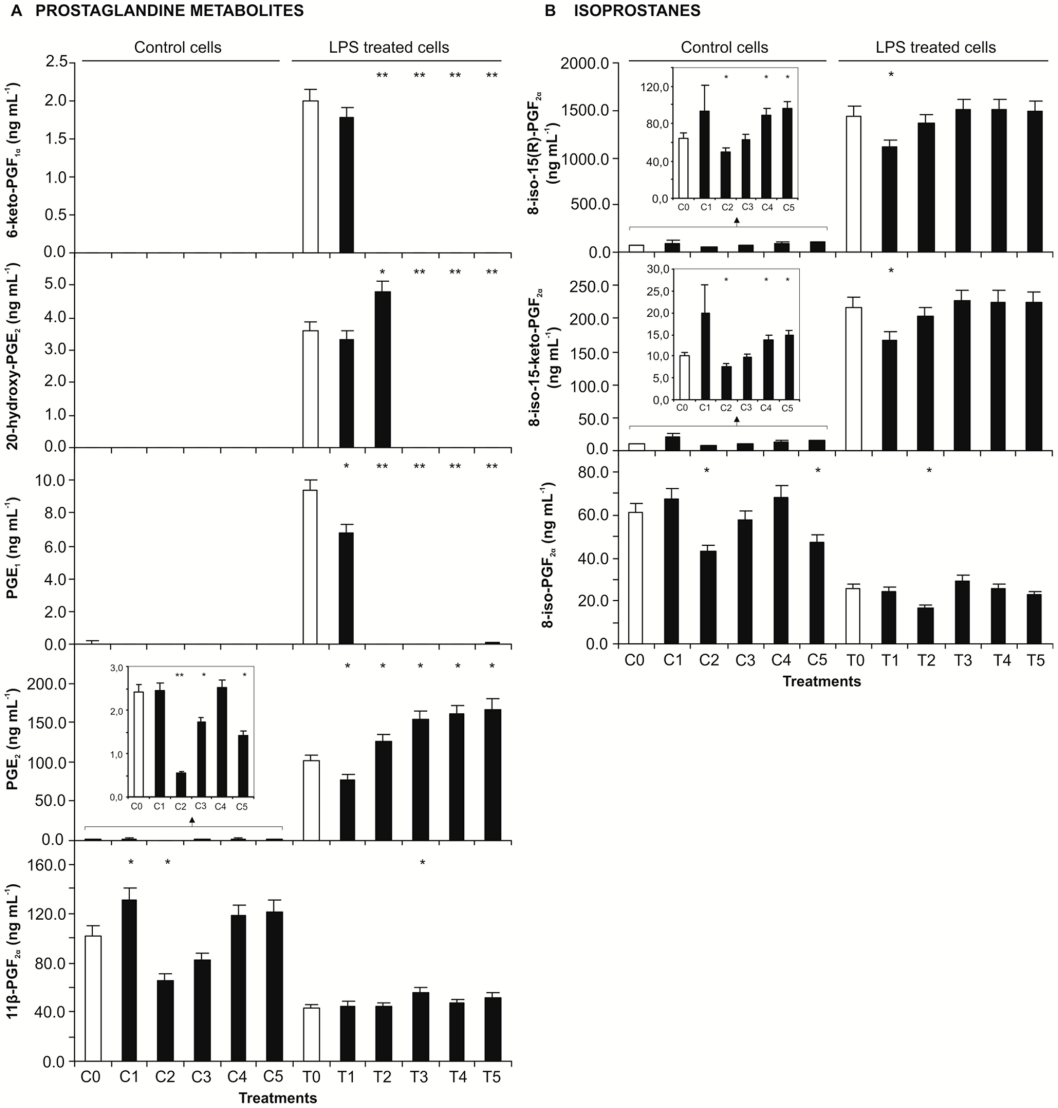
Effect of *E. plantagineum* bee pollen extract pre-exposure in eicosanoids release of LPS-stimulated macrophages. Eicosanoids were determined in the culture media of RAW 264.7 macrophages pre-exposed to the extract for 1 h followed by 8 h co-exposition with 1 mg mL^−1^ of LPS. C group: control group exposed to extract only. T group: extract and LPS co-exposed group. Extract concentrations: C0 and T0: 0; C1 and T1: 0.5 mg mL^−1^; C2 and T2: 1.0 mg mL^−1^; C3 and T3: 2.1 mg mL^−1^; C4 and T4: 4.2 mg mL^−1^; C5 and T5: 8.3 mg mL^−1^. Values show mean ± SEM of two independent assays experiments performed in triplicate. (A) Prostaglandin and prostaglandin metabolites. (B) Isoprostanes. *p<0.05; **p<0.01 compared to the respective control (with or without LPS).

The eicosanoids profile in the culture medium of RAW 264.7 macrophages is shown in Figure 7. Two prostaglandins (PGE_1_ and PGE_2_) and three prostaglandins metabolites (6-keto-PGF_1α_, 20-hydroxy PGE_2_ and 11β-PGF_2α_) were determined (Figure 7A). PGE_1_ release to the medium was increased by LPS exposure and the extract at all concentrations tested was able to decrease the levels of this PG. PGE_1_ returned to the levels observed in the culture medium of non-stimulated cells, for extract concentrations higher than 0.5 mg mL^−1^ (Figure 7A).

Also PGE_2_ release was highly increased by LPS exposure (Figure 7A). Although this prostaglandin was also measured in non-stimulated cells, a 20 fold increase was observed after 8 hours exposure to LPS. The extract decreased PGE_2_ release into the medium at 1 mg mL^−1^ in control cells and at 0.5 mg mL^−1^ in LPS exposed cells. For higher extract concentrations the levels of PGE_2_ in the T group were higher than the ones found in LPS-only exposed cells (*p*<0.05). On the other hand, considerable amounts of 20-hydroxy PGE_2_ were found only in the medium of LPS exposed cells and LPS plus extract at 0.5 and 1 mg mL^−1^. A similar behaviour was observed for 6-keto-PGF_1α_, a PGI_2_ metabolite that was only quantified in the medium of LPS exposed cells and LPS plus extract at 0.5 mg mL^−1^.

Considering 11β-PGF_2α_, the amount of this PG metabolite was higher in the culture medium of non-stimulated cells, compared to LPS-stimulated cells. Particularly in LPS-stimulated RAW macrophages, the extract did not have a significant effect on the release of 11β-PGF_2α_, except when at 2.1 mg mL^−1^.

In what concerns to IsoPs, it was observed that both 8-iso-15(*R*)-PGF_2α_ and 8-iso-15-keto-PGF_2α_ increased with LPS exposure, while the levels of 8-iso-PGF_2α_ decreased (Figure 7B). The effect in the former IsoPs was significantly reduced by the extract only at the lowest concentration tested (*p*<0.05). However, in control cells, the extract at 0.5 mg mL^−1^ significantly increased the levels of 8-iso-15(*R*)-PGF_2α_, 8-iso-15-keto-PGF_2α_, as well as that of the prostaglandin metabolite 11β-PGF_2α_.

Regarding 8-iso-PGF_2α_, the extract at 1 mg mL^−1^ further decreased its level in LPS-exposed cells.

Although PGF_2α_ has been implicated in acute inflammation, in this model of inflammation, tetranor-PGFM, which is PGF_2α_ main metabolite [24], was not detected neither in whole cell extracts nor in the culture medium.

U-44069, an analogue of PGH_2_, thromboxane A_2_ (as its stable analogue U-46619) and 11-dehydrothromboxane B_2_ were not encountered in whole cell lysates or culture medium of macrophages.

The search for 2,3-dinor-6-keto-PGF_1α_ (a PGI_2_ metabolite) and the IsoP 2,3-dinor-8-iso-PGF_2α_ in the cell lysate and culture medium gave negative results.

Comparing the levels of eicosanoids in the culture medium and inside the cell (Figures 6 and 7B and 7C) it can be observed that eicosanoids are mainly secreted into the medium, with the exception of 11β-PGF_2α_ and 8-iso-PGF_2α_ that are present at similar levels inside and outside the cell.

## Discussion

The HPLC-DAD analysis of *E. plantagineum* bee pollen hydromethanolic extract showed its richness in non-coloured flavonoids, being mainly constituted by kaempferol heterosides, some of them acylated with acetate (Figure 2, Table 1). The extract was then assayed for its antioxidant and anti-inflammatory potential in a cellular model taking into account that the absorption of bioactive compounds, extracted from natural matrices and incorporated into supplements or after direct ingestion of whole bee pollen, will occur in some extent, making them bioavailable to cells [25]. Because pollen from *Echium* species were described to be a potential source of pyrrolizidine alkaloids, a class of secondary metabolites with hepatotoxic properties [26], the presence of alkaloids in the extract was screened by the general alkaloid precipitation tests (Dragendorff's, Mayers's and Bertrand's) [27], giving negative results.

The cellular model of LPS-stimulated macrophages is widely used to assess anti-inflammatory activity [5], [11], [17], [18]. It is well known that infection of cells by microorganisms activates the inflammatory response due to recognition by macrophages receptors of LPS present in microorganisms' cell wall: the initial sensing of infection is mediated by innate pattern recognition receptors that are expressed by macrophages dendritic cells and various nonprofessional immune cells. Although inflammation is a protective response of the body to ensure removal of detrimental stimuli, as well as a healing process for repairing damaged tissue, the stimulation of macrophages, initiated by microorganisms (or other agent) can result in the overproduction of inflammatory mediators like PGE_2_ and PGI_2_, which enhance edema formation and leukocyte infiltration by promoting blood flow in the inflamed region. This process can be at the origin of inflammatory diseases like type 2 diabetes, atherosclerosis, and Alzheimer's disease [28], [29].

Before testing in macrophages, the extract was screened for antioxidant activity against ^•^NO and O_2_^•−^ in cell-free systems. These preliminary results revealed that the extract was able to scavenge the sodium nitroprusside-generated ^•^NO (Figure 3). Concerning O_2_^•−^, an antioxidant effect was observed only for the lowest concentrations tested. At higher concentrations, the compounds present seem to participate in the formation of O_2_^•−^ in the PMS/NADH system (Figure 3). The direct interaction of the compounds present in the extract with NBT was excluded because the reduction of NBT to formazan did not occur when the assay was performed in the absence of PMS and NADH. Furthermore, we can also assume that superoxide is not generated by the direct oxidation of kaempferol derivatives by molecular oxygen. So, the apparent pro-oxidant behaviour at higher extract concentrations observed in this evaluating system for O_2_^•−^ scavenging potential can be without biological significance since PMS and NBT are not endogenous molecules. Nevertheless a pro-oxidant behaviour has been previously observed with flavonoids and flavonoid-rich extracts [30], [31].

The anti-inflammatory potential of the extract was assessed *in vitro* using the LPS-stimulated RAW macrophages model. Inflammatory mediators derived from iNOS and COX-2 were measured in an integrated approach.

In relation to ^•^NO, LPS induces its production by iNOS accompanied by L-citrulline formation in stoichiometric amounts [8]. Accordingly, the decrease of the radical was paralleled by the decrease of L-citrulline in the culture medium of LPS-stimulated macrophages exposed to the extract (Figure 5). Given these results, we can state that the extract inhibits iNOS induction by LPS.

Besides ^•^NO, LPS-stimulated macrophages produce PGs as a consequence of COX-2 up-regulation. Attending to the evanescence of primary PGs, we proceeded to the measurement of some PGs metabolites by UPLC-MS. As isoprostanes suffer glucuronidation [23], [32], and UDP-glucuronosyltransferases are present in macrophages [33] an hydrolysis step was included in the eicosanoid extraction protocol to ensure that all eicosanoids found in the macrophages lysate and/or cell culture supernatants were totally determined by UPLC-MS and to avoid underestimations of their concentrations, as we have detailed in previous reports [22], [23].

**Figure 8:**
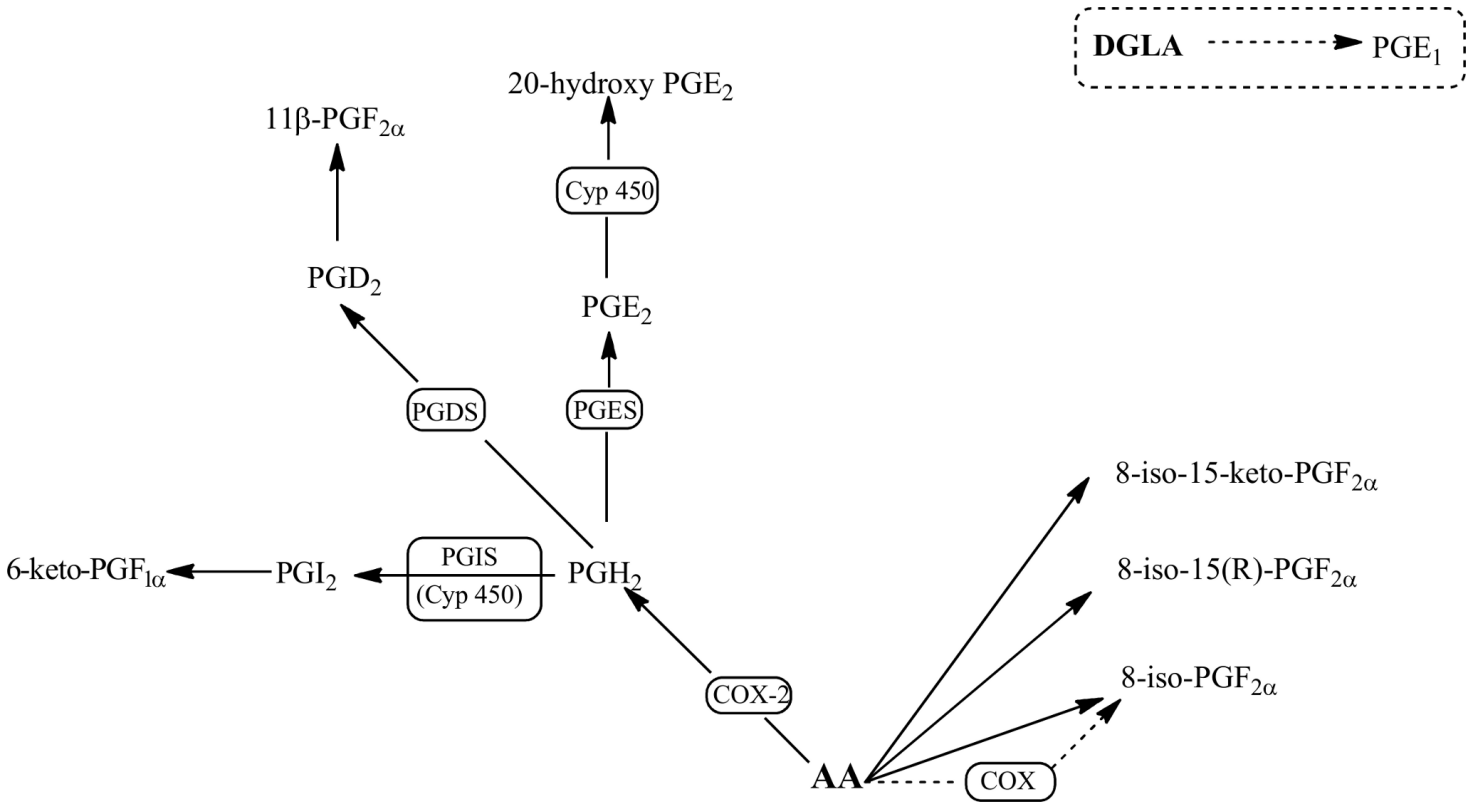
Metabolic pathways of eicosanoids determined in LPS-stimulated macrophages pre-exposed to *E. plantagineum* bee pollen extract. AA, arachidonic acid; COX, cyclooxygenase; Cyp 450, cytochrome P450 enzyme; DGLA, dihomo-γ- linolenic acid; PG, prostaglandin; PGS, PG synthase.

The action of COX on arachidonic acid leads to the formation of PGH_2_, the substrate for the various PG synthases (Figure 8). *In vivo* studies have shown that COX-2 inhibitors reduce PGE_2_ more profoundly than other PGs [34]. PGE_2_ was the prostaglandin with the highest increase both in the whole cell lysate and culture medium of LPS-stimulated macrophages (Figures 6 and 7). The selective increase of this PG in LPS-stimulated rat macrophages is probably due to the up-regulation of PGE_2_ synthase in concert with COX-2 [35], [36].

PGE_2_ undergoes hydroxylation at C-20 catalyzed by cytochrome P450 enzymes, originating 20-hydroxy PGE_2_ [37]. This PGE_2_ metabolite was released to the culture medium of macrophages in response to LPS stimulation, but the extract at concentrations higher than 1 mg mL^−1^ decreased the release of 20-hydroxy PGE_2_ below the quantification limits as observed in the culture medium of non-exposed cells (Figure 7). Therefore, concentrations of the extract >1 mg mL^−1^ were able to neutralize the toxic effect exerted by LPS. This result can be attributed to the known inhibition of cytochrome P450 enzymes by flavonoids [38]. In opposition, the effect of the extract on PGE_2_ was not so pronounced, being more effective at lower concentrations (Figures 6 and 7). Tetranor-PGEM and tetranor-PGAM, PGE_2_ metabolites [10], were not detected.

PGE_1_, a PG derived from dihomo-γ- linolenic acid (Figure 8), was found in the culture medium of LPS stimulated macrophages. The extract was more effective in decreasing the release of PGE_1_ than of PGE_2_ (Figure 7).

Another prostanoid found in the culture medium of cells exposed to LPS was 6-keto PGF_1α_, a PGI_2_ metabolite (Figure 7). PGI_2_ formation is mediated by prostacyclin synthase, a member of the cytochrome P450 monooxygenase family. PGI_2_ is readily hydrolyzed to form the stable 6-keto PGF_1α_ [10]. Interestingly, cells treated with the lowest extract concentration did not significantly alter the concentration of this metabolite, although for all the other extract concentrations 6-keto PGF_1α_ levels were below quantification limits. So, it is possible that the phenolic compounds present in the extract exert an inhibitory effect on prostacyclin synthase, under inflammatory conditions, as seen for the inducible form of NOS.

Thromboxane A_2_ biosynthesis is also catalysed by a member of the cytochrome P450 enzymes and it counteracts PGI_2_ action [10]. The absence of thromboxane A_2_ and its metabolite could be expected, since thromboxane A_2_ formation is mediated by COX-1, although in LPS-stimulated cells COX-2 can also be involved [35].

PGD_2_ has important roles in the mediation and resolution of inflammation. This PG is converted to several metabolites, including 11β-PGF_2α_, which maintains the biological activity of PGE_2_ [39]. 11β-PGF_2α_ was quantified in the cellular lysate and culture medium of control and LPS-exposed cells (Figures 6 and 7). The increase of PGE_2_ in the culture medium of LPS-stimulated macrophages compared to control group was accompanied by a decrease of 11β-PGF_2α_ (Figure 7). PGD_2_ is a structural isomer of PGE_2_, and this can explain the opposite tendency observed for this prostanoid. In un-stimulated macrophages PGD_2_ generation is coupled to the constitutive COX-1 [35]. So, the decrease of 11β-PGF_2α_ in the culture medium of LPS-stimulated cells when compared to control cells can be justified by the preferred production of PGE_2_, which is in agreement with bibliography [35]. This fact can also explain why the extract had no effect in the release of 11β-PGF_2α_ into the medium, since phenolic compounds mainly affect COX-2 [12]. However, the intracellular level of 11β-PGF_2α_ was increased by LPS and a decrease of this metabolite was observed when cells were also exposed to extract (except for the highest concentration) in comparison with cells exposed only to LPS (Figure 6).

As previously said, the bee pollen extract used in this work was characterized by the presence of kaempferol and quercetin derivatives. It is well known that kaempferol inhibits iNOS, COX-2 expression and PGE_2_ formation in LPS-activated RAW macrophages [40]. Also quercetin was found to decrease PGE_2_ in the culture medium of LPS-stimulated RAW macrophages [41], the reduction being much stronger in J774A.1 macrophages [11].

The iNOS inhibitory activity of flavonoids is attributed to the inhibition of NF-κB induction [40]. The more active flavonoids possess a double bond between C-2 and C-3 and a 5,7-dihydroxyl group. The hydroxyl group at C-3 decreases the inhibitory potency [19]. On the other hand, COX-2 inhibition by flavonoids is less studied, but is also partly attributed to NF-κB inhibition. NFκB is involved in COX-2 protein expression in LPS-stimulated J774.1 macrophages and the exposition of these cells to NF-κB inhibitors before LPS challenge abrogates the generation PGE_2_ and 6-keto PGF_1α_ [42]. In fact, 6-keto PGF_1α_ release was inhibited by the extract, except for the lowest concentration tested, while a decrease of PGE_2_ release was only observed for this concentration (Figure 7).

It can be expected polyphenol aglycones to be more potent than their heterosides. For instance, Kim and collaborators showed that isoliquiritigenin was significantly more active than its 4-*O*-glucoside (isoliquiritin) in reducing ^•^NO and PGE_2_ production, as well as protein and mRNA expression of iNOS and COX-2 [12]. This result was explained by the different lipophilicity, which affects the cellular uptake and compatibility with cellular membranes [12]. Also the inhibition of iNOS and COX-2 expression by quercetin was higher than the one of its 3-*O*-rutinoside, rutin [41]. Nevertheless, the decrease of PG observed when cells were exposed to the extract at 0.5 or 0.1 mg mL^−1^ is probably due to the inhibitory action of the flavonol heterosides that characterize the bee pollen extract.

It has been suggested a cross-talk between NOS and COX enzymes [43]. ^•^NO may act through generation of peroxynitrite as the endogenous oxidant needed to create the tyrosyl radical required for catalytic activity of COX enzymes. ^•^NO exerts divergent effects on constitutive and inducible COX isoforms, activating COX-1, but inactivating COX-2 [43]. This may explain why extract concentrations that reduce ^•^NO to lower levels failed to reduce the levels of PGE_2_, the major PG released by LPS stimulated macrophages (Figures 5 and 7).

Conversely, PGE_2_ demonstrated to modulate ^•^NO pathway in LPS-stimulated macrophages [44]. These results were not confirmed by Swierkosz and collaborators, who found that the formation of COX metabolites had no effect on NOS activity, whereas ^•^NO inhibits both COX-2 activity and induction [45].

IsoPs are derived from the free radical-catalyzed peroxidation of arachidonic acid in a COX independent mechanism (Figure 8). Due to the lack of enzymatic control, a series of stereoisomers can be formed [10]. IsoPs are reliable markers of lipid peroxidation *in vivo* and potentially mediate some of the adverse effects of oxidant injury [13]. In this study three IsoPs of the 8-series (containing the nonprostane hydroxyl group at C-8) were encountered, both in the cell lysate and in the culture medium (Figures 6 and 7). In general, LPS treatment leads to an increase of IsoPS, which can be a consequence of the increase of ^•^NO in LPS-exposed macrophages. The excess of ^•^NO production can lead to peroxynitrite formation, which is associated with increased IsoP generation *in vitro* and *in vivo* [46], [47].

8-iso-PGF_2α_ showed a different behaviour, since the release of this IsoP into the culture medium after 8 h exposition to LPS decreased when compared with control cells (Figure 7). 8-iso-PGF_2α_ can be formed *via* COX-2 in monocytes and, as so, it was expected to increase under a pro-inflammatory stimulus [48]. Nevertheless, at this time the level of 8-iso-PGF_2α_ in cell lysate of LPS stimulated cells was higher than in control cells (Figure 6).

IsoPs levels showed a tendency to be increased by extract concentrations above 1 mg mL^−1^, despite the decrease of ^•^NO contents (Figures 5, 6, and 7). This can be attributed to a pro-oxidant behaviour of phenolic compounds when present at high concentrations (observed in the O_2_^•−^ cell-free assay), despite the ability of these compounds to inhibit iNOS induction in macrophages under an inflammatory stimulus [11], [30], [41].

## Conclusions

*E. plantagineum* bee pollen extract contains significant amounts of phenolic compounds, which can exert antioxidant and anti-inflammatory activity. *In vitro* assays demonstrated that the extract is able to scavenge the reactive species ^•^NO and O_2_^•−^, although for the later a pro-oxidant behaviour was observed for the higher concentrations tested. The extract reduces ^•^NO and prostaglandins release in LPS-stimulated macrophages but, in what concerns to prostaglandins, this is true only if the extract concentrations were maintained below certain levels. Conversely, the levels of prostaglandin metabolites derived from cytochrome P450 enzymes, like 20-hydroxy-PGE_2_ and 6-keto-PGF_1α_, were reduced by high extract concentrations. However, under those conditions several isoprostanes were increased in LPS-stimulated and extract-only exposed cells. These results demonstrate that the beneficial anti-inflammatory effects of the extract can be superimposed by a pro-oxidant behaviour. Further *in vivo* experiments are needed to determine the levels of pollen consumption that can constitute a hazard for human health. Nevertheless, the consumption of moderate amounts of pollen or of its bioactive compounds can impart beneficial health effects by reducing the levels of oxidative stress and inflammatory mediators involved in the genesis of many diseases.
